# Clinical applications of next generation sequencing in cancer: from panels, to exomes, to genomes

**DOI:** 10.3389/fgene.2015.00215

**Published:** 2015-06-17

**Authors:** Tony Shen, Stefan Hans Pajaro-Van de Stadt, Nai Chien Yeat, Jimmy C.-H. Lin

**Affiliations:** ^1^Rare Genomics InstituteBethesda, MD, USA; ^2^School of Medicine, Washington UniversitySaint Louis, MO, USA

**Keywords:** next-generation sequencing (NGS), exome sequencing, cancer genomics, clinical genomics, non-coding DNA

## Abstract

This article will review recent impact of massively parallel next-generation sequencing (NGS) in our understanding and treatment of cancer. While whole exome sequencing (WES) remains popular and effective as a method of genetically profiling different cancers, advances in sequencing technology has enabled an increasing number of whole-genome based studies. Clinically, NGS has been used or is being developed for genetic screening, diagnostics, and clinical assessment. Though challenges remain, clinicians are in the early stages of using genetic data to make treatment decisions for cancer patients. As the integration of NGS in the study and treatment of cancer continues to mature, we believe that the field of cancer genomics will need to move toward more complete 100% genome sequencing. Current technologies and methods are largely limited to coding regions of the genome. A number of recent studies have demonstrated that mutations in non-coding regions may have direct tumorigenic effects or lead to genetic instability. Non-coding regions represent an important frontier in cancer genomics.

## Introduction

Cancer, in its many forms, accounted for 8.2 million deaths in 2012 (GLOBOCAN, 2012). The rapid development of DNA sequencing technologies has driven a revolution in our understanding of this highly complex and diverse group of diseases (Devita and Rosenberg, 2012). This review fulfills two purposes. First, this article summarizes the history of massively parallel next-generation sequencing (NGS) in the context of cancer genomics and reviews recent research and clinical applications. Second, we highlight the importance and potential of complete or 100% genome sequencing, i.e., the ability to sequence highly repetitive non-coding sequences beyond the reach of current NGS technologies.

## Background and history

### Sequencing the first cancer exomes and genomes

The first cancer exomes were sequenced soon after the completion of the Human Genome Project in 2001. Compared to whole genome sequencing (WGS), exome sequencing covers only the 1% of the genome that is translated into protein, greatly reducing the technical burden of data collection and analysis. Ley et al. piloted the use of NGS to study the exomes of 140 samples of human acute myeloid leukemia (AML) cells in 2003, identifying 6 previously described and 7 undescribed mutations relevant for AMP pathogenesis. The investigation searched for mutations capable of altering gene function and identified the FLT3 gene as a distinguishing mutant in AML patients (Ley et al., 2003).

The first solid tumor exomes to be investigated were from 11 breast and 11 colorectal cancer tissue samples. Sjoblom et al. identified 189 frequently mutated genes associated with these cancers, most of which were not previously known. Additionally, the investigators found that the average tumor accumulates an average of ~90 mutations over its course of development, though only a subset of mutations contributes to the formation of neoplasms (Sjöblom et al., 2006). A follow-up study revealed varying mutation frequencies for individual genes with few but commonly mutated gene “mountains” and numerous but infrequently mutated gene “hills” (Wood et al., 2007). Despite similarities in the number of mutations for each cancer, the types and locations of these mutations result in distinct cancer subtypes (Wood et al., 2007).

Ley et al. performed the first whole-genome sequencing study on AML cells collected from a single patient. The patient's skin cells were used as a control. The study identified 2 genes known to contribute to tumor progression and 8 known to be present in tumor cells but which have unknown functions. As a proof-of-concept, the study demonstrated the feasibility of WGS as an unbiased tool for the molecular profiling of individual cancers (Ley et al., 2008). Table 1 provides a summary of cancers and gene mutations.

**Table 1:** **Summary of genetic mutations associated with selected cancers**.

**Cancer type**	**Somatic mutations**	**Structural rearrangements**	**Insertions/Deletions**	**Hereditary mutations**	**References**
Melanoma	HRAS, BRAF, RAS, CTNNB1, GNA11, GNAQ, KIT, MEK1, NRAS, MMAC1, PTEN, GRIN2A, PREX2, RAC1, PPP6C, STK19, SNX31, TACC1, ARID2, CDK4, MITF	CDKN2, HMGI-C, RAF	MMAC1, PTEN, BRAF	MAP2K1, MAP2K2, CDK4	Brose et al., 2002; Vogelstein and Kinzler, 2004; Garraway et al., 2005; Wei et al., 2011; Hodis et al., 2012; Vogelstein et al., 2013
Lung Cancer	TP53, KRAS, STK11, EGFR, ALK, BRAF, RAS, AKT1, DDR2, HER2, MEK1, NRAS, PIK3CA, PTEN	RET, ROS1, ALK, EML4, NTRK1	EGFR, FHIT, FRA3B, FGFR1, HER2	EGFR, BRAF, RAS, KRAS, TP53	Brose et al., 2002; Kosaka et al., 2004; Vogelstein and Kinzler, 2004; Soda et al., 2007; Lipson et al., 2012; Vogelstein et al., 2013
Liver Cancer	CTNNB1, GNMT, p57, IN1	GNMT	E1B, GNMT, p57	ARID1A, ARID1B, ARID2, MLL, MLL3, TP53, CTNNB1, EGFR, GNMT, IN1	Biegel et al., 1999; Tseng et al., 2003; Vogelstein and Kinzler, 2004; Vogelstein et al., 2013
Breast Cancer	FBXW7, AKT2, PI3KCA, CCND1, ERBB2, NTRK1, SMAD2, MAP2K4, AKT1, ESR1, FGFR1, FGFR2, HER2, PIK3CA, PTEN	BRCA1, BRCA2, TP53, PIK3CA, CHEK2	BRCA1	TP53, BRCA1, BRCA2, PTEN, AKT1, TP43, TBX3, RUNX1, CBFB	Vogelstein and Kinzler, 2004; Walsh et al., 2006; Stephens et al., 2009; Vogelstein et al., 2013
Colorectal Cancer	CTNNB1, BAX, FBXW7, PI3KCA, FES, KRAS, NRAS, SMAD2, SMAD4, TGFBR1, TGFBR2, MAP2K4, PTNP1, AKT1, PTEN, BRAF, PIK3CA	ALK	APC-L1	FAP, AXIN2, MSH2, MLH1, MSH6, PMS2, HNPCC, MUTYH, PIK3CA, IRS2, IRS4, PTEN, RHEB	Miki et al., 1992; Vogelstein and Kinzler, 2004; Wood et al., 2007; Lipson et al., 2012; Vogelstein et al., 2013
Ovarian	FBXW7, AKT2, ERBB2, TGFBR1, TGFBR2, BRAF, KRAS, PIK3CA, PTEN	ARID1A, BRCA1	MMP-1, BRCA1, BRCA2, MLPA, MAPH	STK11, BRCA1, BRCA2	Kanamori et al., 1999; Sellner and Taylor, 2004; Vogelstein and Kinzler, 2004; Jones et al., 2008; Vogelstein et al., 2013
Pancreatic	KRAS2, N-RAS, MAP2K4, CDKN2A, TP53, SMAD4, BRCA2	TP53, SMAD4, INK4A	CDKN2A, TP53	STK11, CDKN2A	Bardeesy and Depinho, 2002; Montagna et al., 2003; Vogelstein and Kinzler, 2004; Vogelstein et al., 2013
Thyroid	NTRK1, BRAF, KRAS, RET	RET/PTC, ELE1, AKAP9, PTEN, BRAF, TRK	PTEN	RET, NTRK1, BRAF	Fugazzola et al., 1996; Dahia et al., 1997; Cheung et al., 2001; Vogelstein and Kinzler, 2004; Vogelstein et al., 2013

## Discoveries

### Melanoma

Analyzing genomic data from melanoma samples is uniquely challenging because of the high number of passenger mutations caused by ultraviolet light exposure (Hodis et al., 2012). To overcome this, Hodis et al. controlled for UV-induced mutational load by comparing mutated genes-of-interest against a baseline level of intronic mutation. They identified six novel genes: *PPP6C, RAC1, SNX31, TACC1, STK19*, and *ARID2*. Of these genes, *PPP6C, RAC1*, and *STK19* are thought to be potentially targetable. In a WES study of 147 melanomas, Krauthammer et al. identified a P29S mutation *RAC1* in 9.2% of sun-exposed exposed melanomas. Mutations in the active site of *PPP6C*, a serine/threonine phosphatase, were found in 12% of sun-exposed melanomas that already possessed *BRAF* or *NRAS* mutations (Krauthammer et al., 2012).

Mutations in *BRAF, NRAS*, and *KIT* are known to be involved in the pathogenesis of metastatic melanoma. *BRAF* inhibitors are a class of targeted therapeutics approved for treating metastatic melanoma and have demonstrated significantly improved overall survival (Kunz et al., 2013).

### Breast cancer

The Cancer Genome Atlas network sequenced 510 breast cancers exomes, identifying 4 distinct subtypes: luminal A (ER+ and/or PR+, HER2−), luminal B (ER+ and/or PR+, HER2+), HER2-enriched (ER−, PR−, HER2+), and basal-like (ER−, PR−, HER2−). 40% of luminal A tumors possessed a mutated *PIK3CA* gene. *TP53* and *PIK3CA* were mutated in 29% of luminal B tumors. Compared to luminal A or luminal B subtypes, the basal subtype exhibited a more consistent pattern of mutation, with *TP53* mutated in 80% of cases. The HER2-enriched subtype is characterized by *HER2* amplification, found in 80% of these tumors (Koboldt et al., 2012). Basal type tumors are characterized by the highest amount of mutations, while luminal A types generally contain the lowest frequencies of mutations (Wang et al., 2014b). Another study investigated CAG repeat lengths of breast cancer tumor samples to determine the significance of intratumor genetic heterogeneity (ITGH) (Gottlieb et al., 2013). The findings between differing repeat lengths showed that shorter CAG repeats may play protective roles against breast cancer, as opposed to longer repeat lengths, which may contribute to cancer development (Gottlieb et al., 2013). The study demonstrates that merely identifying genetic variations does not provide sufficient understanding of cancer etiology; rather it is necessary to determine frequency and distribution of mutations between cancerous and normal tissues (Gottlieb et al., 2013; Riahi et al., 2014).

## Clinical utility

The information generated by next generation sequencing (NGS) technologies enables clinicians to make improved diagnostic and treatment decisions. For example, breast cancers have traditionally been diagnosed by mammogram, physical exam, and histology. The discovery of *BRCA1, BRCA2*, and other biomarkers introduced genetics as an important consideration (Van De Vijver et al., 2002; Van't Veer et al., 2002). Today, commercially available micro-array-based tests such as OncoType DX and MammaPrint allow more accurate profiling of breast cancers based on genetic biomarkers such as *HER2, ER*, and *PR*, each with their own treatment protocols. In parallel with improved diagnostics, identification of cancer-associated genes has led to the development of molecularly targeted therapies such as trastuzumab, which was among the first therapies specifically targeted to *HER2+* breast cancers. Moreover, sequencing of hundreds of breast tumors has also revealed significant intratumor heterogeneity, reflecting an additional level of complexity for the development of new treatments (Desmedt et al., 2012; Shah et al., 2012).

### Genetic screening

As the cost of NGS continues to approach the $1000 threshold, population-wide genomic screening becomes more likely (Brunicardi et al., 2011). Already, NGS may improve genetic testing in families with histories of high penetrance cancer genes such as *BRCA1, BRCA2, APC*, and *TP53* (Meldrum et al., 2011). Several investigators have tested the Illumina HiSeq platform in detecting *BRCA1, BRCA2*, and *TP53* from a tumor cell line (Morgan et al., 2010; Schroeder et al., 2010). In these studies, NGS analysis identified all known variants in the tumor cell line with sensitivity and specificity greater than traditional diagnostic methods, demonstrating the effectiveness of NGS as a diagnostic tool. More important than the improvement in sensitivity/specificity, the data generated by NGS allows for more sophisticated analysis of gene interactions.

Economical NGS screening will also benefit patients with *de novo* mutations who would not otherwise undergo genetic screening based on family history. In the case of *BRCA* mutations, family history only accounts for 30–50% of mutations (Moller et al., 2007). Additionally, NGS testing allows for testing of genes with a wide range in frequency (Meldrum et al., 2011). Several companies and institutions offer cancer gene panels that screen for over 70 genes (Washington University in St. Louis, University of Washington, Baylor College of Medicine, Ambrygen, Genewiz). While NGS remains too costly for routine sequencing of all individuals, we expect the prevalence of screening to continue increasing as prices decrease.

### Diagnostics and assessment

Currently, NGS-based gene panels are regularly used for cancer diagnostics. For example, the 2015 National Comprehensive Cancer Network guidelines recommend NGS gene panels for patients with hereditary and ovarian cancer who have tested negative for high-penetrance genes (Park et al., 2014). In a study of 141 patients who tested negative for *BRCA1/2*, evaluation by an NGS panel of 40 genes identified 16 patients who have pathogenic variants in 9 non-*BRCA* genes (Kurian et al., 2014). The use of NGS in clinical diagnostics may be separated into three approaches: gene panels, whole exome sequencing (WES), and whole genome sequencing. Among these, gene panels have been in use for the longest period, with more than 16 laboratories in the United States panels for hereditary cancer (Wang et al., 2014a). The diagnostic yields of these panels range from 20 to 51%, which is comparable to that of exome or genome-based methods (Wang et al., 2014a). Recent exome-based diagnostics for mitochondrial respiratory disease and intellectual disability demonstrated diagnostic yields of 60 and 16%, respectively (De Ligt et al., 2012; Taylor et al., 2014). Other evaluations of exome-based diagnostics demonstrated diagnostic yields between 25 and 30% (Yang et al., 2013, 2014; Lee et al., 2014). Genome-based diagnostics are comparatively newer and there are fewer studies that evaluate their use. Studies that evaluated the use of genome sequencing to diagnose intellectual disability and early-onset epilepsy demonstrated a diagnostic yield of 50 and 24%, respectively (Gilissen et al., 2014; Martin et al., 2014). As our understanding of the genome grows, in-depth genome-based diagnostics will have higher diagnostic yield.

### Clinical decision-making and treatment

NGS has enabled investigators to discover and elucidate hundreds of genes involved in cancer, advances that will inevitably reveal novel therapeutic targets. Targeted therapies, a growing group of therapeutic agents with molecular-level specificity have greatly changed the treatment and management for many cancers. Notable examples include imatinib (*BCR-ABL*) and trastuzumab (*HER2*). Targeted therapies have the potential to be more effective and less toxic than traditional chemotherapies for patients suffering from cancer (Tsimberidou et al., 2014). Treatments like EGFR-targeted plasmonic magnetic particles have been shown to be more effective in suppressing lung cancer development and tumor growth by abrogating the G2/M cell cycle phase and inducing apoptosis (Kuroda et al., 2014). Another promising development in targeted cancer therapy is signaling-directed androgen receptor treatment for castration-resistant prostate cancer, which deactivates tumor proliferation pathways and inhibits cancer progression (Bastos et al., 2014). Currently, discoveries on the diagnostics and assessment outpace the development of targeted therapies. As NGS technology matures, the selection of targeted therapies stands to expand greatly as more targets become known.

Due to the easy accessibility of circulating tumor DNA (ctDNA), sequencing ctDNA is another attractive method for analyzing tumor load and treatment effectiveness (Wang and Wheeler, 2014). The mechanism by which ctDNA enters the bloodstream is not well understood, though Gormally et al. propose two mechanisms: release of whole cells followed by lysis and apoptosis (Gormally et al., 2007). Diehl et al. demonstrated that ctDNA measurements in 18 patients with colorectal cancer were a reliable indicator of tumor dynamics (Diehl et al., 2007). In 2014, Lohr et al. developed computational methods to isolate ctDNA sequences from serum. A comparison of their ctDNA sequences to previously sequenced tumor samples revealed 90% of early trunk mutations and 73% of metastatic trunk mutations in the tumor were also found in ctDNA (Lohr et al., 2014). In another study by Murtaza et al. sequencing of ctDNA from patients with breast, lung, and ovarian cancers allowed investigators to track mutations in the tumor genome in a non-invasive manner (Murtaza et al., 2013). Additionally, Dawson et al. demonstrated that the sensitivity of ctDNA surpassed that of other circulating biomarkers (Dawson et al., 2013). These studies suggest that NGS analysis of ctDNA holds great potential for screening, diagnosis, and clinical decision-making.

There remain many challenges for investigators and clinicians. With massive amounts of data generated, healthcare infrastructure needs new strategies for data curation. Additionally, the economic implications of widespread sequencing are not yet known given the complex interplay between healthcare providers, the biomedical industry, insurance companies, and academic research (Buchanan et al., 2013).

## Commercialization

Today, patients may choose between many services and technologies offered by gene sequencing companies for assistance in patient diagnosis and treatment decision processes. Some companies, such as Foundation Medicine and Personal Genome Diagnostics, offer clinical tests like FoundationOne and CancerComplete to help identify the genetic variants in a patient's DNA sample that may be contributing to cancer development.

A number of companies and institutions provide individual gene panel tests for different types of cancers. Cancer-specific kits such as AmbryGen's OvaNext and ColoNext selectively enrich genes-of-interest for ovarian and colorectal cancers, respectively. Other companies and institutions offer similar cancer-specific testing, such as Neogenomics Laboratories, GPS@WUSTL, Emory Genetics Laboratory, ARUP Laboratories, Myriad Genetics, and GeneDx. WES-based tests such as the FoundationOne or FoundationOne-Heme have been developed to be highly sensitive and specific (Frampton et al., 2013). Table 2 provides a summary of commercial services using NGS.

**Table 2:** **Summary and comparison of NGS diagnostic services**.

**Company**	**Product**	**Disease**	**Scope of coverage**	**No. of Genes analyzed**	**Investigates**
Foundation Medicine	Foundation One	Cancer	Panel	236 genes, 47 introns from 19 genes associated with rearrangement	Solid tumors–gene alterations, alteration frequency
Foundation Medicine	Foundation One Heme	Cancer	Panel	405 genes from DNA including 31 introns associated with rearrangement, 265 genes from RNA, fusion genes	Hematologic tumors–gene alteration, alteration frequency, fusion genes
Personal Genome Diagnostics (PGDX)	Cancer Complete	Cancer	Full exome	~20,000	Gene point mutations, copy number alterations (indels), rearrangements
PGDX	Cancer Select	cancer	120 cancer gene panel	120	Point mutations, copy number alterations, rearrangements
Ambry Genetics	Exome Next	Cancer	Exome	~20,000	Mitochondrial genome mutations, sequence variants
Ambry Genetics	BRCA1 and BRCA2 gene sequencing	Breast Cancer	BRCA1 and BRCA2	2	Gene sequencing, deletion and duplication, large rearrangements
GeneDx	XomeDx	Cancer	Full exome	~20,000	Exon analysis
GeneDx	XomeDx Plus	Cancer	Exome	20,000 plus mitochondrial sequencing	Combined test, mitochondrial genome sequencing and deletions
GeneDx	XomeDx Slice	Cancer	Exome	targeted test	Targets regions of the exome or specific genes–variant search
GeneDx	Comprehensive cancer panel	Cancer	Panel	29	Gene sequence, deletions/duplications, gene mutations, nucleotide substitutions
NeoGenomics Laboratories	EGFR Mutation Analysis	NSCLC	EGFR exons 18-21	1	Mutations on target exons, duplications/deletions
NeoGenomics Laboratories	NeoSITE Melanoma	Cancer	Panel	5	Copy number variants, duplications/deletions
NeoGenomics Laboratories	FISH for non-small cell lung cancer	NSCLC	Panel	2	Rearrangements, fusions
NeoGenomics Laboratories	Colorectal Cancer panel	Colorectal Cancer	Panel	2	KRAS and BRAF mutations, mismatch repair defects, microsatellite instability at 5 target loci
Caris	MI Profile	Cancer	Panel	47	Somatic mutations in solid tumors
Myriad Genetics	BRCA Analysis	Breast and Ovarian Cancer	BRCA1, BRCA2	2	Gene mutations
Quest Diagnostics	OncoVantage	Solid tumors	Panel	34	Point mutations, indels
GPS@WUSTL	Comprehensive Cancer Gene Set Analysis	Cancer	Panel	42	Point mutations, indels
Arup Laboratories	Gastrointestinal hereditary cancer panel	Gastrointestinal cancer	Panel	15	Targeted capture of coding exons and intron/exon junctions–sequenced for mutation detection, deletion/duplication analysis

## Future directions

### Single molecule sequencing

While NGS has undoubtedly driven a revolution in genetics, current technologies face certain limitations. First, the amplification of DNA libraries may result in differential expansion of certain regions of the genome. This introduces the problem of biased reads. Second, epigenetic modifications are lost during amplification. Third, the short read lengths generated from current NGS platforms requires a reliance on a reference genome and robust alignment algorithms to produce sequences from raw NGS data (Mardis, 2013). Lastly, the entire sequencing process from sample preparation to computational construction of the sequence takes days to weeks (Liu et al., 2012).

Single molecule or third generation sequencing systems address shortcomings present in current NGS platforms. Single-molecule real-time sequencing (SMRT, Pacific Biosciences) and nanopore sequencing (Oxford Nanopore) are perhaps the most well-known. Conceptually, both of these systems detect nucleotide incorporation onto a single DNA strand in real time. SMRT uses a system of fluorescent signals to monitor nucleotide incorporation while nanopore detects voltage across a lipid bilayer as as DNA is ratcheted across an α-hemolysin nanopore (Eid et al., 2009; Liu et al., 2012). Sequencing a single strand eliminates the need for amplification, reducing sequencing bias present in NGS platforms. Monitoring systems can also be tuned to detect epigenetic changes. Read lengths are also significantly longer compared to current NGS systems, with SMRT reads in the range of ~5000–6000 bp (English et al., 2012; Chin et al., 2013). Long read lengths allow for rapid *de novo* sequencing of organisms without reference genomes as well as the sequencing of highly repetitive regions. Additionally, simplified preparation and sequencing in real time with nucleotide incorporation significantly shortens run times. SMRT runs can be completed in just 1 day (Liu et al., 2012).

Single molecule sequencing systems are beginning to make an impact in cancer genomics and are poised to play a larger role moving forward. While SMRT has been available since 2011, nanopore sequencing has yet to see widespread use. SMRT has been used to resequence AML patients to identify a marker (*FLT3* with internal tandem duplication) associated with poor prognosis (Smith et al., 2012). Kleinman et al. used SMRT to elucidate a distinct DNA methylation pattern and epigenetic alteration program in embryonal brain tumors (Kleinman et al., 2014). In addition to the ability to study genetic and epigenetic changes undetectable by NGS, single molecule sequencing also holds great potential for sensitive diagnostics. Sequencing results comparable to NGS reads may be obtained from as little as 500 pg of starting DNA (Raley et al., 2014). In late 2013, Pacific Biosciences announced a large-scale collaboration with Roche to produce diagnostics tools based on SMRT (Pacific Biosciences Investor Relations, 2013).

### Diagnostic yield

As the field moves forward to understand a wider range of genetic abnormalities, there must be a concurrent shift toward 100% genome sequencing for diagnostics. However, current genome-based diagnostics still face several challenges. Dewey et al. analyzed 12 healthy genomes for 56 clinically significant genes and found that the best platform (Illumina) was only able to sequence 51 out of 56 genes with adequate coverage (Dewey et al., 2014). This is a technical hurdle that we hope will be overcome with improved technologies and bioinformatics. Genome-based diagnostics are also inherently limited by the inability to sequence the whole genome, with approximately 8% of the genome not sequenced. This challenge may only be overcome with 100% genome sequencing. Improvement in the diagnostic yield of genetic testing can only occur given a solid foundation of complete gene coverage. Given that the relatively low diagnostic yields of current strategies, we submit that the goal of 100% genome sequencing will be clinically significant.

### 100% genome sequencing

Current findings suggest that a variety of defects in non-coding regions can contribute to neoplastic pathogenesis (Chmielecki and Meyerson, 2014). Huang et al. identified a pattern of mutations in the promoter region of telomerase reverse transcriptase, demonstrating that somatic mutations in non-coding regions can have a direct tumorigenic effect (Huang et al., 2013). Zhu et al. demonstrated that genomic instability following the loss of *Brca1* in mice may be due to de-repression of satellite DNA transcription. The authors propose that tumor suppressor functions of *BRCA1* primarily stem from its role as a regulator of heterochromatin (Zhu et al., 2011). Ting et al. identified a pattern of satellite transcript overexpression in epithelial cancers of the lung, kidney, ovary, colon, and prostate, proposing that heterochromatic alterations may serve as a biomarker for cancer (Ting et al., 2011). These findings suggest that non-coding regions may be a rich source of new insights into tumorigenesis.

Complete genomic sequencing may also reveal epigenetic changes in cancer that were previously inaccessible due to limitations in the coverage and resolution of last-generation technologies The epigenetics of nucleosomes for example, have eluded investigation in the past, but new protocols utilizing NGS which enable genome-wide studies of nucleosome positioning at high sensitivity might allow networks of chromatin modulation to finally be probed (Wei et al., 2012). Using NGS, Kim et al. characterized the instability of microsatellite regions in colorectal and endometrial cancers, showing that this genetic instability affects nucleosome positioning (Kim et al., 2013). Studies of the epigenetics of cancer have already produced surprising findings, such as the discovery that regions of DNA hypomethylation in breast cancer cells are paradoxically, silenced by the formation of repressive chromatin domains (Hon et al., 2012), and the discovery that histone modifications drive pediatric glioblastoma (Rheinbay et al., 2012). Still more protocols that utilize NGS for studying epigenetic changes are in the pipeline: A recently published method of deciphering histone post-translational modifications (PTMs) using DNA-barcoded nucleosome libraries, is enabled in large part by the speed and low-cost of high throughput DNA sequencing (Nguyen et al., 2014). A thorough understanding of chromatin regulation however, will require not just knowledge of DNA base sequences, but also an understanding of how DNA segments interact with histones and their regulatory elements. To this end, biophysical models, such as a recently published model by Chertsvy and Teif on the electrostatics of histone-DNA interactions (Cherstvy and Teif, 2014), are essential. In another modeling study, Beshnova et al. demonstrate that although major satellite repeats in mice encode preferences for nucleosome positioning, nucleosome positioning is not rigid, and can alternate between two separate types of positioning (Beshnova et al., 2014).

## Final comments

In this article, we discussed the past, present, and future of DNA sequencing in cancer genomics. As advancements in sequencing technology enabled investigators to study cancer genomes in greater breadth and depth, the field has produced novel insights into tumor pathogenesis, identified clinically useful biomarkers, and developed increasingly precise diagnostics and targeted therapeutics. However, it is important to note that our understanding of the genome is limited, with <5000 of 23,000 known (Park et al., 2014). The clinical usefulness of genomic sequencing requires advancement in our knowledge of the genome and bioinformatic systems to process genetic data—advancements that would be built on a foundation of 100% genome sequencing.

### Conflict of interest statement

The authors declare that the research was conducted in the absence of any commercial or financial relationships that could be construed as a potential conflict of interest.
